# Enhanced motor imagery of digits within the same hand via vibrotactile stimulation

**DOI:** 10.3389/fnins.2023.1152563

**Published:** 2023-06-09

**Authors:** Vadivelan Ramu, Kishor Lakshminarayanan

**Affiliations:** Neuro-Rehabilitation Lab, School of Electronics Engineering, Vellore Institute of Technology, Vellore, Tamil Nadu, India

**Keywords:** motor imagery, electroencephalography (EEG), event-related desynchronization (ERD), vibrotactile, brain computer Interface (BCI)

## Abstract

**Purpose:**

The aim of the present study is to evaluate the effect of vibrotactile stimulation prior to repeated complex motor imagery of finger movements using the non-dominant hand on motor imagery (MI) performance.

**Methods:**

Ten healthy right-handed adults (4 females and 6 males) participated in the study. The subjects performed motor imagery tasks with and without a brief vibrotactile sensory stimulation prior to performing motor imagery using either their left-hand index, middle, or thumb digits. Mu- and beta-band event-related desynchronization (ERD) at the sensorimotor cortex and an artificial neural network-based digit classification was evaluated.

**Results:**

The ERD and digit discrimination results from our study showed that ERD was significantly different between the vibration conditions for the index, middle, and thumb. It was also found that digit classification accuracy with-vibration (mean ± SD = 66.31 ± 3.79%) was significantly higher than without-vibration (mean ± SD = 62.68 ± 6.58%).

**Conclusion:**

The results showed that a brief vibration was more effective at improving MI-based brain-computer interface classification of digits within a single limb through increased ERD compared to performing MI without vibrotactile stimulation.

## Introduction

Motor imagery (MI) refers to mental “rehearsal” of defined motor sequences without the concomitant movement of the actual muscles (Pfurtscheller and Neuper, 1997; Andrade et al., 2017). Motor imagery activates many of the same cortical areas as motor execution (ME), such as primary motor cortex, premotor cortex, and somatosensory cortex (Ehrsson et al., 2003; Sauvage et al., 2013; Lorey et al., 2014). MI furthermore has been shown to induce cortical plasticity resulting in motor performance improvements in healthy subjects (Hashimoto and Rothwell, 1999; Jackson et al., 2003; Stinear et al., 2006; Lebon et al., 2012) as well as spinal-cord injured patients (Mateo et al., 2015). Specifically, one of the commonly used markers of MI for MI-based brain computer interface (BCI) classification is the event-related desynchronization (ERD) in the mu (8–12 Hz) and beta (13–30 Hz) frequency band elicited during imagination of motor movements (Pfurtscheller and Neuper, 1997; McFarland et al., 2000; Jeon et al., 2011). MI-based BCI training is largely used for individuals seeking to improve their motor performance (Hong et al., 2017).

MI training largely employs imagining movements with the right and left hand or feet since they exhibit clear neurophysiological differences and consequently are easier to classify in BCI applications (Lotte et al., 2018). However, such simple MI have limited use for patients with reduced motor performance, since neurofeedback from the affected hand is more essential than using both hands in rehabilitation (Benzy et al., 2020; Dahms et al., 2020). Therefore, complex MI training employing tapping of fingers from a single limb has been employed to replace conventional simple MI in such cases (Ma et al., 2019).

The complex MI, however, has a few limitations. In stroke survivors, the affected hand has greatly reduced MI ability due to the motor deficit (de Vries et al., 2011) making the implementation of single limb complex MI difficult. Even healthy subjects exhibited handedness-based cortical differences (Tecchio et al., 2006). Studies have shown that MI abilities differ between dominant and non-dominant hands (Maruff et al., 1999), with the dominant hand showing better MI performance compared to the non-dominant hand (Guillot and Collet, 2010). Such asymmetrical cortical activations with handedness or motor deficits may reduce BCI classification accuracy. Furthermore, MI classification robustness within a single limb may be affected due to similar neurophysiological patterns between the various movements with the given limb.

Integrating tactile stimulation with MI has shown promise in improving MI performance. A study by Mikula et al. (2018) showed that during a reaching task, the proprioception of the hand showed improvement when provided with additional tactile information through vibrotactile stimulation. Furthermore, electrophysiological studies have found that MI combined with sensory stimulation greatly improved the motor imagery vividness (Mizuguchi et al., 2015), and enhanced cortical response for the imagined hand (Mizuguchi et al., 2009, 2013) but not for the non-imagined hand (Mizuguchi et al., 2012). Furthermore, a recent study (Shu et al., 2017) found that integrating MI tasks with unilateral tactile stimulation in the non-dominant hand and paretic hand of healthy individuals and stroke survivors, respectively, significantly increased the contralateral cortical activations for the stimulated hand during MI tasks, but had no effect on activation patterns during MI tasks of the non-stimulated hand. However, it is unknown if such improvement in MI performance via tactile stimulation can be seen for complex MI involving multiple digits from a single limb.

The purpose of this study is to determine the effect of vibrotactile stimulation during repeated complex motor imagery of multiple finger movements using the non-dominant hand on MI performance. To achieve this, we examined brain activities using electroencephalography (EEG) during MI with versus without vibrotactile stimulation. Machine learning techniques were applied to discriminate MI tasks performed by the index, middle, and thumb fingers of the non-dominant hand based on their sensorimotor responses. It was hypothesized that vibrotactile stimulation combined with MI would increase the ERD response and greater task discrimination during finger movement tasks within the non-dominant hand compared to without vibrotactile stimulation.

## Methods

### Subjects

Ten healthy right-handed adults (4 females and 6 males) with ages ranging between 19 and 38 years participated in the study. All subjects verbally disclosed that they had no history of upper limb injury or musculoskeletal or neurologic disorders. All subjects had no prior experience with motor imagery. The protocol was approved by the Vellore Institute of Technology Review Board. Subjects read and signed a written informed consent form before participating in the experiment.

### Procedure

The mu- and beta-band ERD at the sensorimotor cortex was evaluated with and without vibrotactile stimulation applied on the index, middle, or thumb digit pads prior to when subjects performed motor imagery tasks using the index, middle, or thumb digits, respectively. Subjects performed the motor imagery sessions with and without sensory stimulation on two separate days with at least 2 weeks of washout period in between the sessions.

#### Sensory stimulation

**Figure 1:**
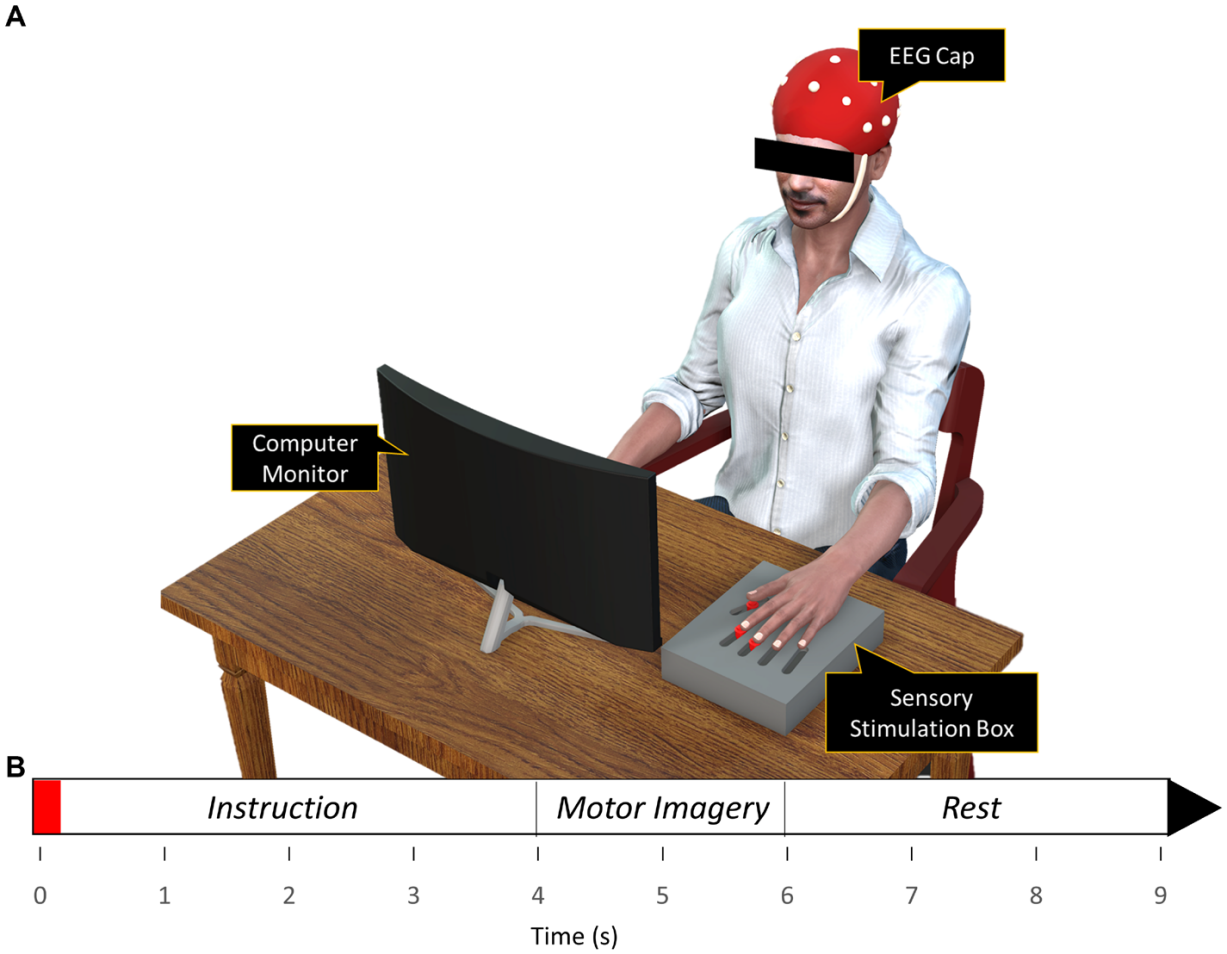
**(A)** Experimental setup **(B)** Timeline of the finger movement task. Subjects observed the action via the animation displayed in the computer monitor while performing motor imagery of the action displayed on the monitor. The red region on the timeline signifies the 150 ms period of vibration applied during the with-vibration condition.

The sensory stimulation was applied via vibration through a flat vibration micro motor (Sunrobotics, Gujarat, India). A sensory stimulation box (Figure 1) was constructed which contained three vibration motors for sensory stimulation of individual digits (index, middle, and thumb). Adjustable sliders were built-in to align the individual vibration motors with individual digit pads. The vibration motor was fed with a white-noise signal filtered between 0-500 Hz and so generated a white-noise vibration that varied randomly between 0-500 Hz. The white-noise vibration was chosen to account for the difference in stimulating frequency between the subjects. During the sessions with the vibrotactile stimulation, the vibration was applied on the corresponding finger briefly for 150 ms at the start of each trial prior to MI performance.

#### EEG recording

The EEG signals were obtained using an Allengers’ Virgo EEG system (Allengers Medical Systems, Chandigarh, India). The system included an EEG cap with 20 electrodes (FP1, FPz, FP2, F7, F3, Fz, F4, F8, T3, C3, Cz, C4, T4, T5, P3, Pz, P4, T6, O1, O2) following the international 10–20 system with FPz and Fz electrodes serving as ground and reference, respectively. The cap was placed on the scalp of each subject after each electrode site was cleaned and adequate application of conductive gel to keep the impedance under 5 kΩ and obtain high quality EEG signals.

#### Experimental design

A 3D left hand was modeled and animated in Blender software (Blender Foundation, Amsterdam, Netherlands). The non-dominant left hand was animated to perform finger movement tasks using the three digits, namely the index, middle, and thumb. Unity game engine (Unity Technologies, San Francisco, CA, United States) was used to gamify the hand and its animation inside a 3D virtual environment. The animation was displayed on a computer monitor for the subjects to observe while performing MI of the same task being shown on the monitor.

The experiment was conducted in a quiet room with minimal environmental distractions. Subjects were positioned comfortably in a chair with their arms placed on the arm rests of the chair. To reduce motion artifacts during EEG recording, subjects were instructed to avoid any movement including eye blinking. The experiment consisted of two motor imagery conditions, namely the with and without sensory stimulation (with-vibration and without-vibration) with each condition consisting of three blocks corresponding to the three digits with which the MI was performed (Index, Middle, and Thumb). A single block had five sessions consisting of 10 trials of MI per session with adequate rest provided between sessions. In total, each subject performed 50 trials for each of the three digits per vibration condition making it a total of 300 trials per subject.

A single trial in the with-vibration condition had a text reminding the subject which of the three digits was being tested. A brief vibrotactile stimulation lasting 150 ms was then applied on the corresponding digit at the finger pad at the start of the trial. The stimulation was followed by 4 s waiting period instructing the participant to be ready and then at the 4-s mark from the start of the trial, the animation showed the digit being tested pressing down on a button, holding the position for 2 s, and then releasing the button to come back to the initial position. The animation was followed by a 3-s rest. Subjects were asked to observe the task animation and imagine kinesthetically the same movement by forming an impression with the same digit being tested performing the button pushing task shown in the animation. A trial run was performed to make the subjects comfortable with imagining the task at the pace it was performed by the 3D model. The without-vibration condition was similar to the with-vibration condition with the exception being no vibrotactile stimulation was applied at the beginning of the trial.

The two sensory stimulation conditions were performed on two separate days with at least 2 weeks in between the sessions to wash out any residual learning effects from the previous session. The order of the sensory stimulation conditions was counterbalanced, with five subjects randomly picked from the ten subjects to perform the without-vibration first and the remaining five performed the with-vibration condition first. The order of the digits was randomized for each subject and each session. During the entire experiment, the EEG signals were recorded continuously at 250 Hz.

### EEG analysis

#### Pre-processing

ERD and digit-discrimination analysis was performed using MATLAB (The MathWorks, Natick, MA) with ERD analysis performed using EEGLAB toolbox and discrimination analysis performed using an artificial neural network available in the Neural Network toolbox in MATLAB. The EEG signals were bandpass filtered between 0.5–50 Hz to remove line noise and re-referenced to a common average reference. Artifacts were removed by performing Independent component analysis (ICA) using the ADJUST algorithm (Mognon et al., 2011). The cleaned data was separated into epochs ranging from −1,000 ms to 8,000 ms relative to the start of each trial.

#### ERD analysis

The C4 electrode was chosen for ERD analysis because it corresponds to the contralateral right sensorimotor cortex activity for the MI performed with the left hand digits. Time-frequency analysis was performed to obtain event-related spectral perturbations (ERSP) that revealed a change in power in the mu- and beta-band during the performance of the motor imagery tasks. ERSP was calculated per trial epoch and baseline corrected relative to the pre-stimulus interval (−1,000 to 0 ms) for each trial. Individual ERSPs were then averaged across subjects to obtain an average ERSP. Mu- and beta-band power changes were calculated by averaging the amplitude values within the mu frequency band (8–12 Hz) and beta frequency range (13–30 Hz) respectively.

To examine the effect on MI performance, repeated measures ANOVAs were conducted on the task-related ERD. Specifically, the ERD obtained by averaging the ERSP across one-second (4,500 ms to 5,500 ms) within the two-second MI period (4,000 ms to 6,000 ms) during which the subject imagined performing the finger movements. The ERD was obtained for the mu- and beta-bands separately. A two-way repeated-measures ANOVA was conducted for each frequency band with the independent variables being the vibration condition (with vs. without), and digit (Index, Middle, and Thumb). Post-hoc Bonferroni tests were used for all pairwise comparisons. The statistical analysis of the data was performed using SigmaStat 4.0 (Systat Software Inc., San Jose, CA, United States). An α level of 0.05 was considered for statistical significance.

#### Discriminant analysis

An artificial neural network (ANN) was constructed using the nrptool module, to evaluate the neural activity discrimination of the three digits. The accuracy rate obtained from the ANN would indicate the accuracy with which the neural network was able to classify the three digits for each sensory stimulation condition. The classes were Index, Middle, and Thumb and so a three-class classifier was used. The toolbox utilizes a two-layer feedforward network that has a learning procedure based on the scaled conjugate gradient backpropagation algorithm. Feature extraction was performed on the EEG data from the channels Fz, F4, Cz, C4, Pz, and P4 to extract discriminating features associated with the three digits while performing MI. The six electrodes were chosen to encompass the entire contralateral sensory and motor area. An one-second time period between 4,500 ms to 5,500 ms in the epoch when MI was performed and ERD occurred was extracted from each trial from each of the six electrodes. The extracted ERSP data were then averaged across mu- and beta-band combined (8–30 Hz) for each electrode and concatenated. The concatenated features were then fed into the ANN which had an input layer with 150 neurons (N) corresponding to the 50 trials per digit, a hidden layer with 22 neurons (H), and an output layer with 3 neurons (M) corresponding to three digit classes. The number of neurons assigned to the hidden layer was calculated using the following formula (Fadiyah and Djamal, 2019).


$$\sqrt{N\;x\;M}$$

A “training set” of 70% of the data was randomly selected. 15% of the remaining data was held back and used as “validation data” to validate the model. The remaining 15% was chosen as “testing data” and used to evaluate the model. The neural network training was repeated 20 times to minimize the influence of random fluctuations from the training set during each iteration. The accuracy rates from the 20 runs were then averaged to obtain the final accuracy rate. A paired *t*-test was then performed to compare the accuracy rates between sensory stimulation conditions (with-vibration vs. without-vibration).

## Results

We conducted repeated-measures ANOVAs to study the differences between the vibration conditions. The ANOVA’s presumptions regarding the ERD results were initially confirmed. There were no clearly distinguishable outliers found. According to the Shapiro–Wilk normality test, the normality was not violated for any of the groups (*p* > 0.05), and the data had a normal distribution. When the variances of the variations between experiment conditions are equal, this is known as sphericity. This assumption was tested using the Brown-Forsythe test, and the outcomes showed equal variance (*p* > 0.05).

**Figure 2:**
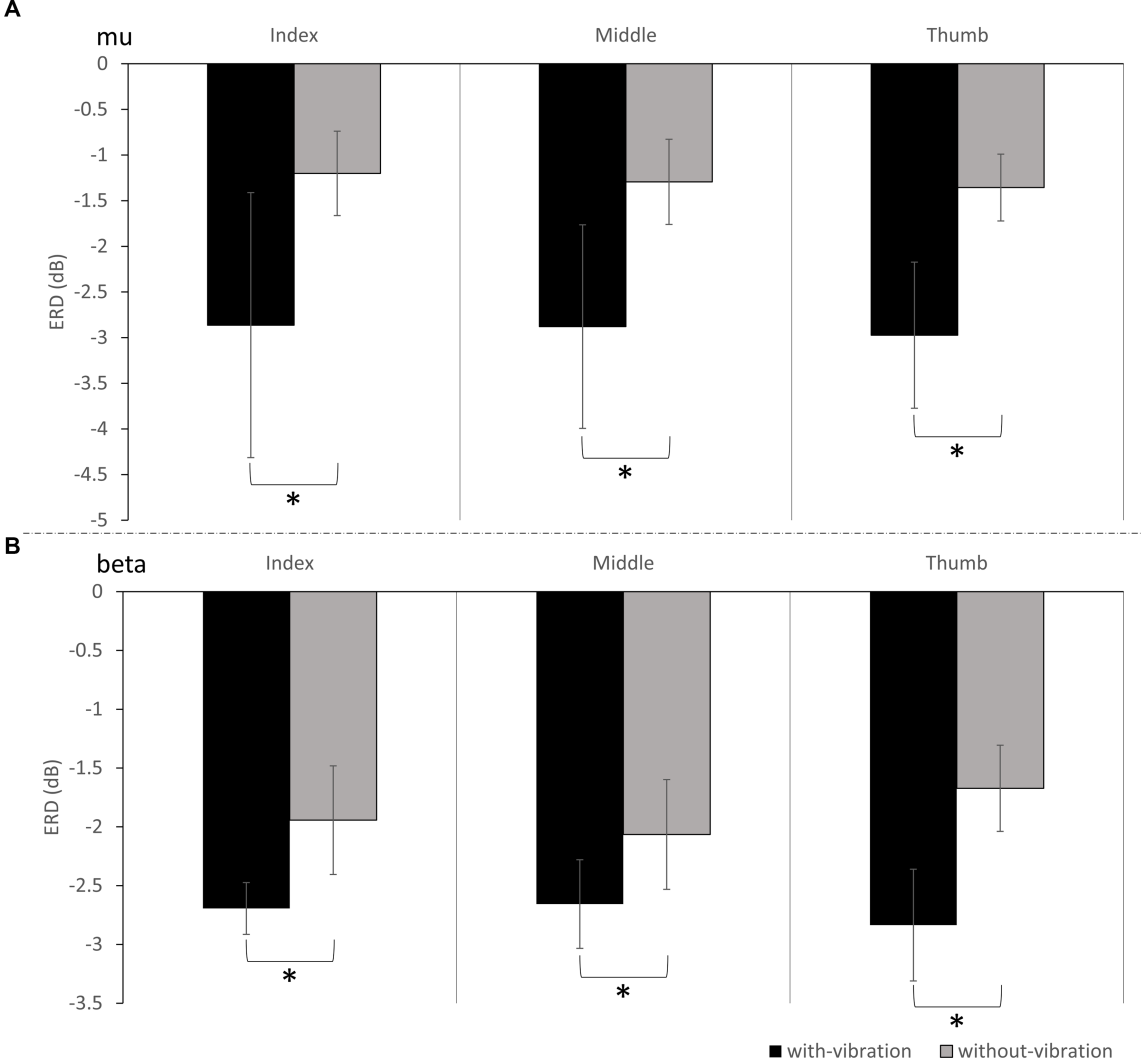
The grand average ERD (mean ± SD) of all subjects for with-vibration and without-vibration in **(A)** Mu-band and **(B)** Beta-Band. A significant difference between the vibration conditions was found for the index, middle, and thumb digits for both bands (^*^ denotes *p* < 0.05).

ERD during motor imagery was compared between the vibration conditions. In the mu-band, repeated measures ANOVA showed ERD significantly differing between the vibration conditions (*p* = 0.003) but not by digit (*p* = 0.767). No interactions were found to be significant (*p* = 0.977). Specifically, the ERD power was lower in with-vibration compared to without-vibration (mean ± SD = −2.90 ± 0.53 dB for with-vibration, mean ± SD = −1.28 ± 1.11 dB for without-vibration). Post-hoc tests revealed that the ERD was significantly different between the vibration conditions (Figure 2A) for the index (*p* = 0.002), middle (*p* = 0.003), and thumb (*p* = 0.003).

**Figure 3:**
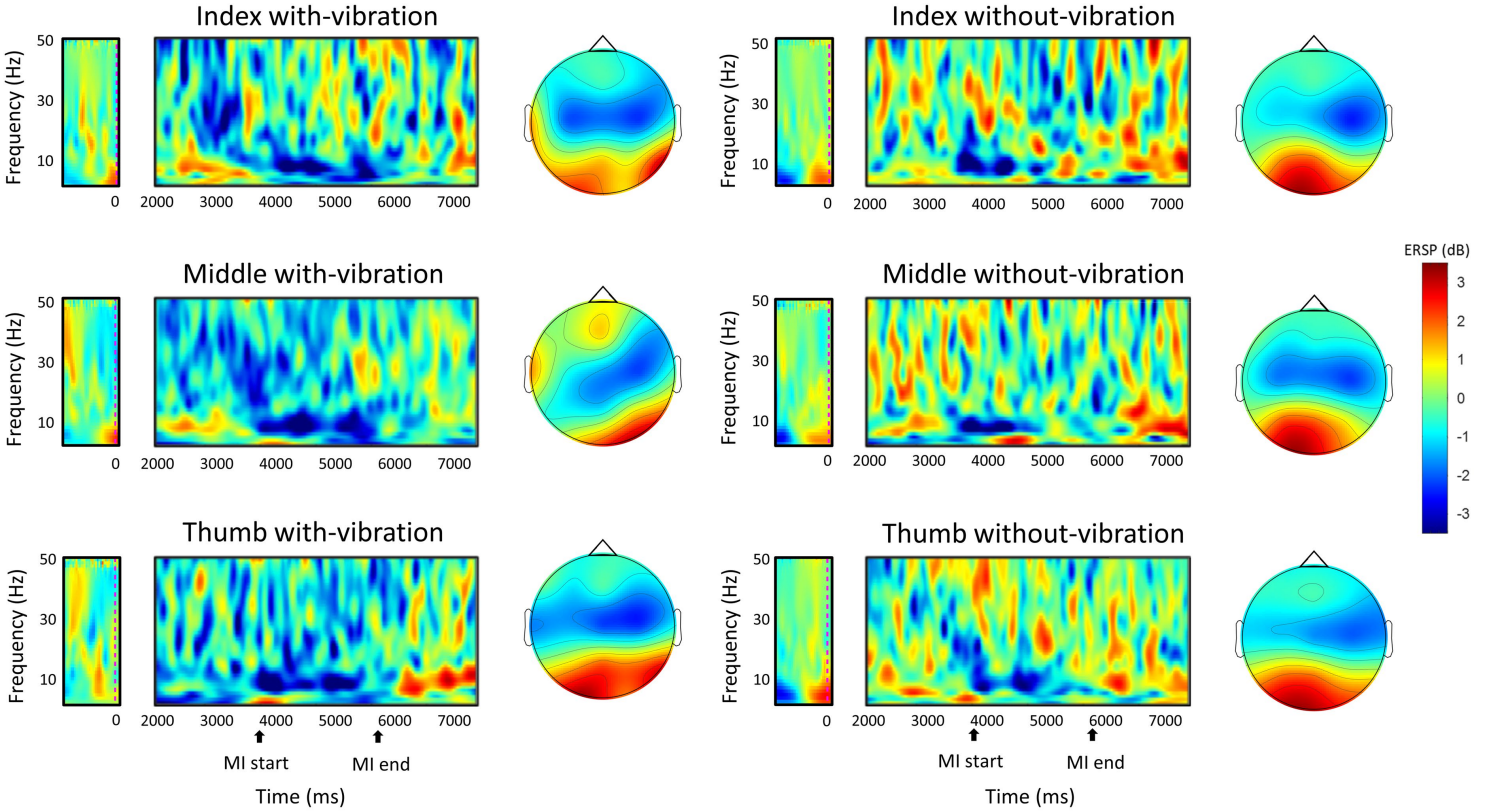
The grand-average ERSP in the time-frequency domain during motor imagery of the left-hand digits and the spatial distribution of the grand-average ERD within the mu and beta band (8–30 Hz) between 4,500 ms to 5,500 ms during the MI task period. The MI start at 4000 ms indicates the start of the animation cue of button-pushing which lasts for two seconds.

Repeated measures ANOVA analysis in the beta-band demonstrated that there was a significant difference in ERD between vibration conditions (*p* < 0.001), while no significant difference was observed between digits (*p* = 0.451). A significant interaction between the vibration condition and digits were found (*p* = 0.032). Specifically, the ERD power was lower with-vibration compared to without-vibration (mean ± SD = −2.72 ± 0.36 dB for with-vibration, mean ± SD = −1.89 ± 0.45 dB for without-vibration). Further post-hoc tests revealed that there was a significant difference in ERD between the vibration conditions (Figure 2B) for the index (*p* < 0.001), middle (*p* = 0.002), and thumb (*p* < 0.001). The MI-induced cortical activations in the time-frequency domain and the spatial distribution of the ERD for the three finger movement tasks with-vibration and without-vibration are shown in Figure 3.

**Figure 4:**
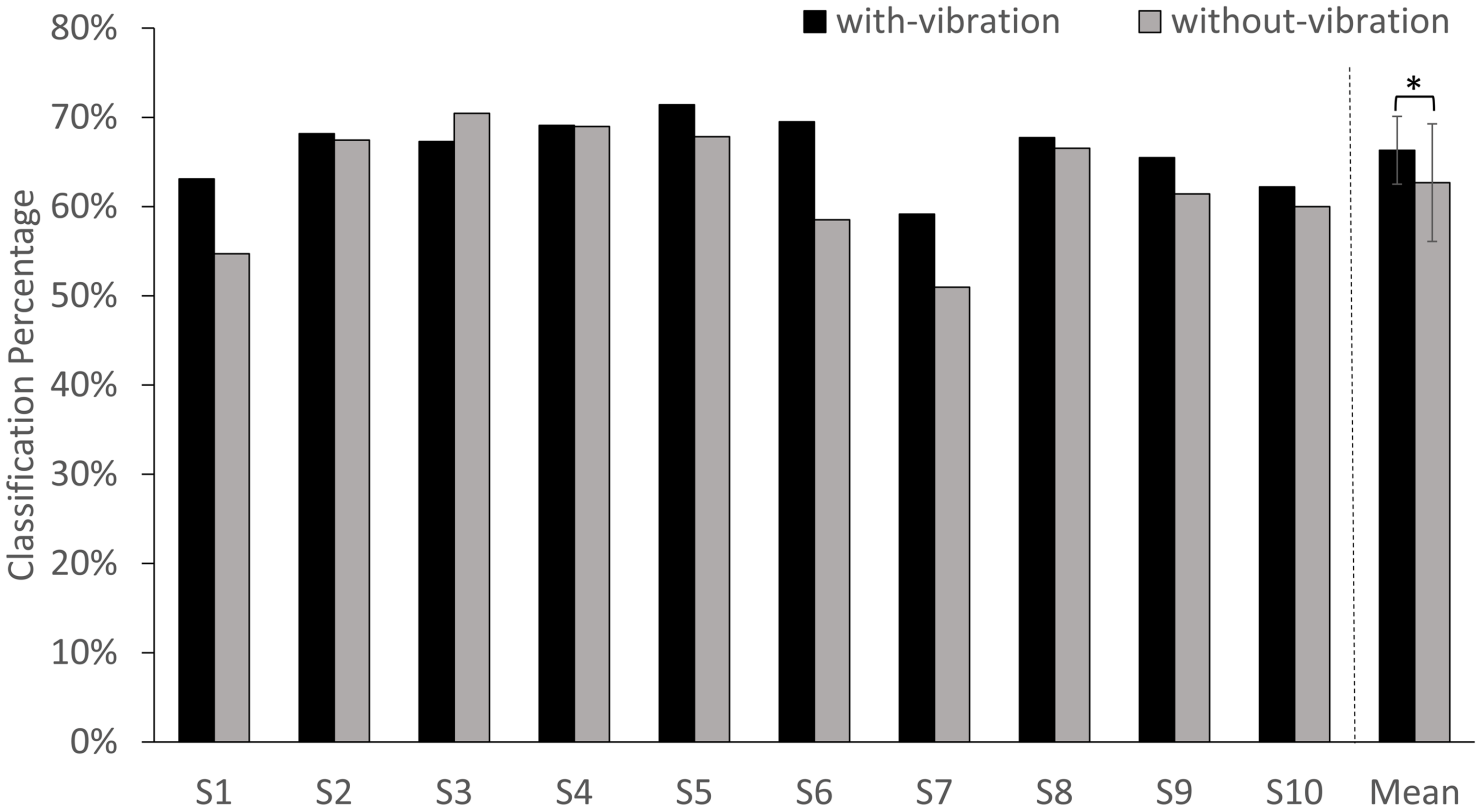
BCI-classification accuracy percentage for each subject and the average of all subjects. Error bars represent the standard deviation (SD). ^*^ denotes *p* < 0.05 using paired *t*-test.

**Figure 5:**
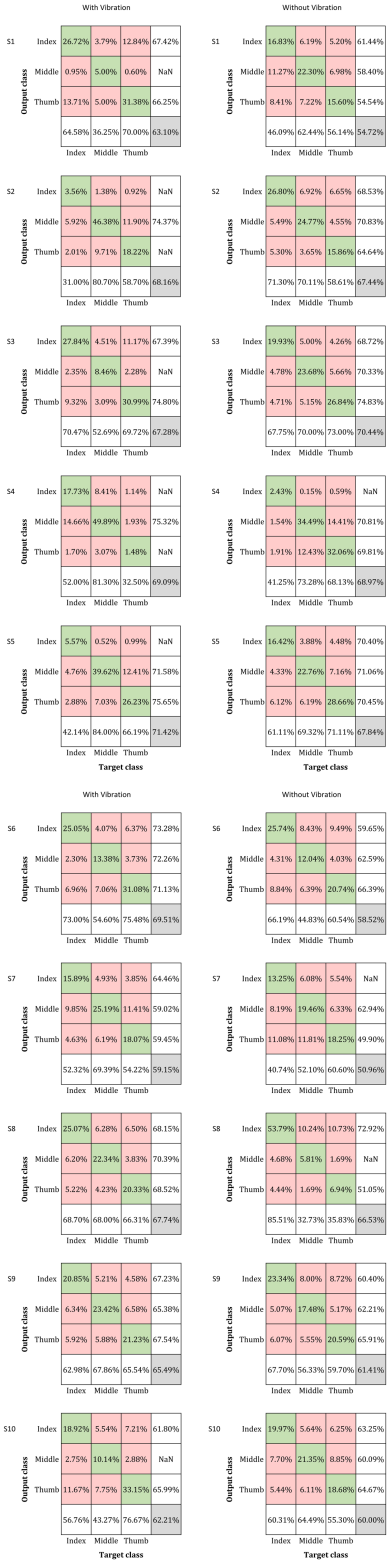
Confusion matrix for the classification of the three digits under the with- and without-vibration conditions for each subject. The rows correspond to the output classes while the columns correspond to the target classes (Index, Middle, and Thumb for both classes). The percentage of correctly classified inputs are shown in the diagonal green boxes while the incorrectly classified entries are in the off-diagonal red boxes. The green and red boxes contain the percentage of the total number of observations that were correctly classified and misclassified, respectively. The column in the far right of the plot displays the precision or positive predictive value for all the correctly classified examples in each class. Similarly, the row at the bottom of the plot shows the percentages of correctly classified examples for each class, referred to as the recall or true positive rate. The grey box in the right end corner shows the overall classification percentage which is the sum of the diagonal entries.

The classification accuracy percentage for with-vibration and without-vibration are shown in Figure 4 and the confusion matrices are shown in Figure 5. A paired *t*-test was performed to evaluate the statistical difference between the classification accuracies from the vibration conditions. The results showed that digit classification accuracy with-vibration (mean ± SD = 66.31 ± 3.79%) were significantly higher (*p* = 0.02) than without-vibration (mean ± SD = 62.68 ± 6.58%).

## Discussion

In this study, we investigated whether tactile stimulation at the digit pad before motor imagery involving the same digit would improve BCI performance, and in particular whether this would lead to a higher ERD. The ERD power during motor imagery in the mu- and beta-bands and the artificial neural-network based digit discrimination results from our study provides evidence that a brief vibrotactile stimulation at the index, middle, and thumb digit pads right before performing motor imagery involving the respective digits does influence the EEG power in the sensorimotor area via ERD response in both the mu and beta frequency bands and MI-BCI classification of the digits compared to without stimulation. Specifically, subjects in our study displayed a higher ERD response in both the mu- and beta-bands and higher digit classification percentage with vibrotactile stimulation compared to no stimulation. Based on the ERD and digit discrimination results from our study, it has been confirmed that sensory stimulation via a brief vibration is more effective at improving MI-BCI classification of digits within the same limb through increased ERD compared to performing MI without stimulation.

The spatial distribution of the ERD activity in the mu and beta frequency bands (8–30 Hz) showed a predominantly contralateral activation for all the digits in both vibration conditions (Figure 3). With mu-band activity reflecting stimulus-related neuronal response (Forschack et al., 2017) and beta-band activity reflecting motor tasks (Khanna and Carmena, 2015), the enhancement of ERD in the both the mu- and beta-bands in the contralateral sensorimotor area during MI following a vibrotactile stimulation could possibly be through the influence of the sensory stimulation on the sensorimotor cortex.

The increase in contralateral sensorimotor region cortical activity with tactile stimulation seen in the current study are similar to previous studies (Yao et al., 2013; Shu et al., 2017; Liburkina et al., 2018). Although the above mentioned studies had a constant vibrotactile stimulation applied to the subjects’ limb while they performed MI, in the current study the stimulation was short (150 ms) and was applied prior to the MI performance. Such an improvement in MI performance and BCI classification with a short stimulation has advantages over constant stimulation where subjects might develop numbness to the stimulation and that might affect the MI performance.

Tactile stimulation has been reported to improve proprioception of the hand (Rizzolatti et al., 1998; Mikula et al., 2018), while proprioception has been shown to enhance corticospinal excitability during MI (Vargas et al., 2004). With proprioception and tactile sensation sharing the same population of posterior parietal neurons during high-level spatial representations (Rizzolatti et al., 1998), it is possible for the sensory stimulation to enhance the ERD response by reaching the sensorimotor cortex and influencing the discharge of the corticospinal cells as documented by previous studies (Lemon, 1981; Cheney and Fetz, 1984). It is acknowledged that the brain combines several sensory information to build an internal representation of its surroundings (Ernst and Bülthoff, 2004; Knill and Pouget, 2004). Motor imagery is believed to rely on this internal representation to predict the future sensory and motor states of the body during motion imagination (Gentili and Papaxanthis, 2015; Nicholson et al., 2018). Such sensory and motor predictions can be enhanced by previous real sensory stimulation (Rulleau et al., 2018). In our study, the vibrotactile stimulation of the digits might provide the required source of sensory information to enhance the internal representation of the digit via proprioception thereby improving the ERD response from the digit while performing MI.

The vibrotactile stimulation not only affected EEG power, but also aided in improving the classification of digits within a given limb using a neural network. Using vibrotactile stimulation during MI to decode brain signals classified the envisioned movement better than using MI without vibration, indicating that the stimulation improved the MI process. Our results show that vibrotactile stimulation enhanced the oscillatory rhythm associated with imagined movement in the same manner as skilled practice did. A high classification accuracy for digits within a single limb has implications in brain computer interface and controlling prosthetic arms. Although repeating motor imagery in both vibration conditions elicited ERD and digit discriminability, the present study showed that when a vibrotactile stimulation is provided to the digit-pad prior to motor imagery with the same digit, this approach can lead to even better performance in MI-BCI.

The experimental design of the current study employed both action observation (AO) and MI. AO + MI has been shown to elicit a larger desynchronization (Berends et al., 2013). To ensure that subjects performed both AO and MI concurrently, clear instructions were given to the subjects to imagine the observed movement kinesthetically. Based on several studies that were reviewed by Vogt et al. (2013) it has been recommended that AO and MI training should be combined and used simultaneously and should not be seen as mutually exclusive means of treatment.

The current study has a few limitations. First, the sample size was small, and subjects displayed some variation between performances of each other that may have affected the statistical power. Thus, the results from this study should be interpreted carefully. Second, neural networks require memory heavy computations and far larger samples for training. Obtaining a large BCI database for finger movements is challenging owing to differences in experimental conditions across various other related studies. Future studies will focus on developing a deep learning framework through advanced data reconstruction methods. We aim to achieve high performance BCI in real-world environments.

## Conclusion

In summary, the current study showed that a brief vibrotactile stimulation applied to the fingertip prior to motor imagery led to an increase in finger movement related ERD activity, indicating activity of the sensorimotor cortex, and a greater digit discrimination within a single given limb.

## Data availability statement

The original contributions presented in the study are included in the article/supplementary material, further inquiries can be directed to the corresponding author.

## Ethics statement

The studies involving human participants were reviewed and approved by Institutional Ethics Committee VIT. The patients/participants provided their written informed consent to participate in this study.

## Author contributions

VR and KL designed the study. VR recruited the subjects and collected data from the subjects. VR and KL analyzed the data. VR and KL wrote the first draft of the manuscript with KL contributing to revisions. KL is the guarantor of this work and, as such, had full access to all the data in the study and takes responsibility for the integrity of the data and the accuracy of the data analysis. All authors contributed to the article and approved the submitted version.

## Funding

This research was supported by the Department of Science and Technology, India (Grant number SRG/2021/000283).

## Conflict of interest

The authors declare that the research was conducted in the absence of any commercial or financial relationships that could be construed as a potential conflict of interest.

## Publisher’s note

All claims expressed in this article are solely those of the authors and do not necessarily represent those of their affiliated organizations, or those of the publisher, the editors and the reviewers. Any product that may be evaluated in this article, or claim that may be made by its manufacturer, is not guaranteed or endorsed by the publisher.

## Abbreviations

MI: Motor Imagery
ME: Motor Execution
EEG: Electroencephalography
BCI: Brain-Computer Interface
ERD: Event-related Desynchronization
ANN: Artificial Neural Network
ERSP: Event-related Spectral Perturbation
AO: Action Observation
